# Experimental and clinical tests of FDA-approved kinase inhibitors for the treatment of neurological disorders (update 2024)

**DOI:** 10.37349/eds.2025.1008116

**Published:** 2025-07-01

**Authors:** Hassan Aliashrafzadeh, Dewey Liu, Samantha De Alba, Imad Akbar, Austin Lui, Jordan Vanleuven, Ryan Martin, Zhang Wang, Da Zhi Liu

**Affiliations:** 1 Department of Neurology, University of California, Davis, CA 95616, USA; 2 Ayub Medical College, Abbottabad 22020, Pakistan; 3 Department of Neurological Surgery, University of California, Davis, CA 95616, USA; 4 Institute for Psychedelics and Neurotherapeutics, University of California, Davis, CA 95616, USA; 5 Department of Chemistry, University of California, Davis, CA 95616, USA; 6 Mirnova Therapeutics, Inc., Davis, CA 95618, USA

**Keywords:** Kinase inhibitors, aberrant cell cycle, clinical trials, preclinical experiments, neurological disorders

## Abstract

Since our previous summary of the 74 FDA-approved kinase inhibitors in clinical and preclinical trials for non-cancerous neurological treatment, the US FDA has approved 13 additional kinase inhibitors since early 2022. This update incorporates new evidence for the now 87 FDA-approved kinase inhibitors in clinical and preclinical trials for the treatment of non-cancerous neurological disorders. By the end of October 2024, nearly all 87 FDA-approved kinase inhibitors have been tested in various animal models of non-cancerous neurological disorders, with twenty entered into clinical trials and six used for off-label treatments of neurological conditions in humans. Considering the challenges posed by intellectual property (IP), legal considerations, and limited blood-brain barrier (BBB) permeability, which may restrict some FDA-approved kinase inhibitors from effectively targeting the central nervous system (CNS), we further discuss the feasibility of designing novel proprietary analogs with enhanced BBB penetration to improve their therapeutic potential in neurological disorders. The new drugs typically retain full IP rights and remain costly; while repurposing kinase inhibitors may provide effective and affordable treatments for non-cancerous neurological disorders.

## Introduction

We developed an original concept of “Aberrant Cell Cycle Disease (ACCD)”, revealing that two major types of diseases (cancers and neurological diseases) share the same mechanism of “aberrant cell cycle re-entry” [1]. The aberrant cell cycle is implicated in both the unlimited proliferation of tumor cells in cancers and the post-mitotic death of mature neurons in non-cancerous neurological disorders [1–3]. Considering that aberrant cell cycle re-entry is manifested as oncogenic kinase activation and/or tumor suppressor activation, the cell cycle arrest (e.g., inhibiting oncogenic kinase, elevating tumor suppressors) that is widely practiced for cancer treatment can be leveraged for the treatment of non-cancerous neurological disorders.

Given human genome encodes ~500 protein kinases and a single kinase often has tens of artificial/synthetic inhibitors, there are likely thousands of synthetic kinase inhibitors. Among those, the FDA has approved a total of 87 kinase inhibitors, mostly for cancer therapy by the end of October 2024, with 74 approved by 2021 and 13 thereafter (i.e., abrocitinib, capivasertib, others) (Supplementary material). Unlike cancer drug development, the advancement of neurologically effective drugs for human use has seen limited success, despite neurological disorders being life-threatening and impacting millions of people worldwide each year [4–6]. Utilizing the ACCD concept, we previously reviewed the 74 kinase inhibitors that the FDA had approved by 2021 [2], summarizing their clinical and preclinical applications for neurological disorders and assessing their potential for repurposing in non-cancerous neurological treatments.

In this update, we incorporate new evidence of using the FDA-approved kinase inhibitors for non-cancerous neurological treatment. In addition to intellectual property (IP) and legal matters, the poor blood-brain barrier (BBB) permeability of some kinase inhibitors limits the repurposed use for neurological treatment, except for acute brain injuries [e.g., traumatic brain injury (TBI), intracerebral hemorrhage (ICH), subarachnoid hemorrhage (SAH)] that are characterized by immediate BBB disruption. To overcome these barriers, this review also discusses the strategy of structure modification of FDA-approved kinase inhibitors to synthesize new compounds of full IP and improved BBB permeability for the treatment of non-cancerous neurological disorders. We searched each individual kinase inhibitor with each subtype of neurological disorder in the databases (https://pubmed.ncbi.nlm.nih.gov; https://clinicaltrials.gov) for published preclinical experiments, clinical trials, and off-label use of the 87 FDA-approved kinase inhibitors for neurological treatments. Of note, non-FDA-approved kinase inhibitors and neurological cancers (e.g., glioblastoma, brain metastasis from peripheral cancers, neuroblastoma) are beyond the scope of this review.

## Kinase, kinase inhibitors, and FDA-approved kinase inhibitors and indications

Kinases, existing universally in various species, have predominantly been thought as oncogenes involving tumorigenesis for many decades [7, 8], in spite of the opposite evidence that shows several kinases [e.g., protein kinase C (PKC), mitogen-activated protein kinase kinase 4 (MAPKK4/MEK4), and death-associated protein kinase 3 (DAPK3)] function as tumor suppressors [7]. Kinases catalyze various reactions of phosphorylation where the high-energy molecules (e.g., ATP) donate phosphoryl groups to substrate molecules [9, 10], with approximately 13,000 human proteins having phosphorylation sites [11]. The transfer of the phosphoryl group from one molecule to another is a fundamental process in many aspects of metabolism, gene regulation, signal transduction, and others [12]. Regarding target substrates, human protein kinases are classified into three types: serine-threonine kinases (STKs), tyrosine kinases (TKs), and dual specificity kinases (STKs/TKs). According to the presence or absence of transmembrane receptor structure, TKs are further divided into receptor TKs (RTKs) and non-RTKs (NRTKs).

There are an estimated thousands of synthetic kinase inhibitors, as the human genome encodes ~500 protein kinases while a single kinase often has tens of artificial/synthetic inhibitors. For example, Src kinase has an increasing list of its synthetic inhibitors: dasatinib, bosutinib, saracatinib, WH-4-023, ENMD-2076, PP1, PP2, pelitinib, ponatinib, PP121, tirbanibulin, SU6656, repotrectinib, UM-164, apatinib, eCF506, elzovantinib, DGY-06-116, 1-NM-PP1, TPX-0046, TL02-59, other.

As for the non-FDA-approved kinase inhibitors, it was reported that bruton TK (BTK) inhibitors (i.e., evobrutinib, fenebrutinib, orelabrutinib, remibrutinib, tolebrutinib) had been tested in clinical trials for treatment of multiple sclerosis (MS) [13]. In addition, a number of non-FDA-approved kinase inhibitors have shown efficacy for the treatment of neurological disorders in experimental models, such as Src family kinase (SFK) inhibitors (PP2, saracatinib) [14–18], rho-associated protein kinase (ROCK) inhibitor (Y-27632) [19–21], extracellular signal-regulated kinase (ERK) inhibitor (PD98059) [22], cyclin-dependent kinases (CDKs) inhibitors (flavopiridol, roscovitine) [23–27], and others. Therefore, many kinase inhibitors (whether FDA-approved or not) can be used to treat both cancers and neurological disorders, which in turn strongly supports the ACCD concept that cancers and neurological disorders share similar underlying pathophysiological mechanisms.

## Clinical trials of FDA-approved kinase inhibitors for neurological treatment

Many of the FDA-approved kinase inhibitors have been tested in clinical and preclinical trials for neurological disorders, though none of them have been approved by the FDA for neurological treatments (Supplementary material). Acalabrutinib, a BTK inhibitor, is currently undergoing investigation in a phase II clinical trial (NCT05065554) to assess the safety and efficacy of its combination with rituximab or other CD20 antibodies for individuals suffering from neuropathies associated with immunoglobulin M monoclonal gammopathy of undetermined significance (IgM-MGUS) or Waldenstrom macroglobulinemia (WM). Alpelisib, an inhibitor of PI3K, is in an ongoing phase II clinical trial (NCT05577754) in pediatric and adult patients with megalencephaly-capillary malformation polymicrogyria syndrome (MCAP).

Baricitinib, classified as a JAK inhibitor, has finished a phase II trial (NCT03921554) and is presently being evaluated in a phase II/III trial (NCT04517253) focused on determining the safety and efficacy of baricitinib for Aicardi-Goutieres syndrome (AGS), a genetic disorder that results in profound mental and physical disabilities in infants. This drug was studied in a phase I/II trial (NCT05792462) assessing the efficacy and safety of baricitinib in neuromyelitis optica spectrum disorders (NMOSD). In another phase III trial (NCT06631287) in patients with long COVID, baricitinib was studied to determine if it can improve neurocognitive function, measures of physical function, quality of life, post-exertional malaise, the effect of breathlessness on daily activities, post-COVID-19 symptom burden, and biomarkers of inflammation and viral measures. The effect of baricitinib administration on the outcome of participants with moderate and severe traumatic ICH/contusions was studied in a phase II clinical trial (NCT06065046). In another trial (NCT04378621), the anti-inflammatory effect of baricitinib was investigated on neuropsychiatric symptoms and morphological changes in the brain caused by rheumatoid arthritis (RA). Other ongoing trials include phase II/III trials (NCT06548802) assessing the efficacy and safety of baricitinib in patients with pulmonary injury after ICH, and a phase II study (NCT05452564) evaluating the efficacy and safety of baricitinib for the reduction of human immunodeficiency virus (HIV) in the central nervous system (CNS). In addition, the role of this drug in CNS mechanisms of anhedonia and psychomotor slowing in depressed people with HIV was investigated in a phase II trial (NCT05849038). Lastly, a phase I/II trial (NCT05189106) assessed the safety of baricitinib in Alzheimer’s disease (AD) and amyotrophic lateral sclerosis (ALS). There is also a case report describing positive effects of baricitinib for the treatment of ocular myasthenia gravis (MG) in a 58-year-old woman who received baricitinib for alopecia areata [28]. The effects of baricitinib, empagliflozin, linagliptin, and telmisartan on cardiovascular autonomic neuropathy in type 1 diabetes were studied in a randomized, crossover trial [29]. The drug bosutinib, known for its inhibitory effects on Src and BCR-ABL, has been the focus of multiple clinical trials addressing various neurodegenerative disorders. Among these trials is a phase I study (NCT04744532) evaluating its application in ALS, along with another ongoing phase I trial (NCT02921477) that investigates its potential benefits for dementia and mild cognitive impairment. Moreover, a completed phase II trial (NCT03888222) has specifically targeted Lewy body dementia (LBD).

In a phase II trial (NCT04079179), Cobimetinib, known for its role as a MAPKK/MEK inhibitor, has been researched for its safety and efficacy in addressing histiocytic disorders that could potentially lead to neurodegeneration. Dasatinib, which acts as an inhibitor of BCR-ABL, Kit, Src, EphA2, EGFR, and PDGFR, has been evaluated in combination with quercetin, a flavonoid recognized for its anti-inflammatory and antioxidant characteristics, in multiple completed trials (NCT04063124 phase I and II, NCT05422885 phase I and II) and is currently under investigation in ongoing trials (phase I and II in NCT04785300, phase II in NCT04685590) for the management of mild cognitive impairment and AD [30, 31]. Extensive clinical trials have investigated Everolimus, an agent that acts as an inhibitor of both FKBP and mTOR, in relation to different types of acute brain injuries, as well as neurodegenerative and neurodevelopmental disorders. Such instances include the completion of NCT03198949 (a phase II trial) of Everolimus for epilepsy and focal cortical dysplasia (FCD), and another completed phase I/II trial (NCT02991807) of Everolimus for hamartoma tumor syndrome. A phase II trial (NCT01997255) that is presently underway is assessing the safety and efficacy of Everolimus for patients experiencing seizures linked to Sturge-Weber syndrome. A total of twelve studies, which include both finished and current trials, are examining the safety and efficacy of Everolimus for patients suffering from tuberous sclerosis complex (TSC), which is commonly connected with FCD, cognitive disabilities, autism, intractable seizures, self-injurious behavior, and a range of other neurocognitive challenges (NCT01070316, NCT01713946, NCT02451696, NCT01929642, NCT01289912, NCT02962414, NCT01730209, and NCT01954693), and a terminated phase II clinical trial study (NCT00857259) evaluated the safety profile and therapeutic efficacy of Everolimus, used alone or alongside ranibizumab, in patients affected by neo-vascular age-related macular degeneration. Imatinib, which acts as an inhibitor of Kit, BCR-ABL, and PDGFR, has been studied in various acute brain injury conditions as well as neurodegenerative diseases. A phase III clinical trial (NCT03639922) evaluated the addition of Imatinib to standard acute stroke treatment, focusing on its potential to enhance functional recovery. Furthermore, a phase II clinical trial (NCT02363361) is examining the safety, absorption, and tolerability of Imatinib in individuals with cervical spinal cord injury (SCI). A separate phase II clinical trial (NCT03674099) is currently evaluating Imatinib as an innovative treatment for MS, assessing its effectiveness in comparison to methylprednisolone, which is the standard treatment for relapses of MS. Additionally, there was an intended phase I clinical trial (NCT00403156) that aimed to explore the application of Imatinib in the treatment of choroidal neovascularization; however, this study has been retracted. Nilotinib functions as a kinase inhibitor, targeting the activities of PDGFR, BCR-ABL, and DDR1. It has undergone investigation into various neurodegenerative disorders through clinical trials. Currently, a phase II trial (NCT04002674) is exploring Nilotinib’s effects on patients with dementia with Lewy bodies, focusing on pharmacokinetics, tolerability, biomarkers, and safety. Also, a phase I trial (NCT03764215) involved administering Nilotinib to individuals diagnosed with Huntington’s disease (HD). The study evaluated biomarkers, including levels of phosphorylated tau, alongside functional outcomes. Currently, a phase III clinical trial (NCT05143528) is underway to assess the safety and effectiveness of Nilotinib in early AD patients, utilizing two distinct dosages. A phase II clinical trial (NCT02947893) investigated the effectiveness of Nilotinib in individuals diagnosed with AD. The study specifically measured the impact of Nilotinib on cell death using various cell markers. Additionally, the concentrations of amyloid in the brain were evaluated through positron emission tomography (PET) imaging. Furthermore, there are three trials, both completed and ongoing, that focus on the effects of Nilotinib in patients with Parkinson’s disease (PD) (NCT03205488, NCT02954978, and NCT02281474). Pazopanib, an inhibitor of PDGFR α/β, FGFR 1/3, VEGFR 1/2/3, Itk, Lck, Fms, and Kit, has been tested in multiple completed or terminated trials (NCT01154062, NCT00659555, NCT00612456, NCT00463320, NCT01072214, NCT01134055, NCT01051700, NCT01362348, and NCT00733304) for macular degeneration. Pirtobrutinib, a BTK inhibitor, was studied in a phase II clinical trial to assess its efficacy and safety in participants with MS (NCT06104683), though the trial has been withdrawn. Regorafenib, a VEGF kinase inhibitor, successfully passed phase I trial for neovascular age-related macular degeneration. The phase IIa trial study (NCT02222207) was terminated as its results did not meet the effectiveness of the current standard of care [32].

Sirolimus, which functions as an mTOR inhibitor and was first used as an immunosuppressant in kidney transplants, has been incorporated into various clinical trials targeting neurological and psychiatric conditions, including epilepsy (NCT03486366, NCT03646240), cerebral aneurysms (NCT04141020), age-related macular degeneration (NCT02732899, NCT01445548, NCT00766649, NCT01675947, NCT00712491, NCT00304954, NCT02357342, and NCT00766337), AD (NCT04629495, NCT06022068, and NCT04200911), ALS (NCT03359538), frontotemporal dementia (NCT04408625), MS (NCT00095329), PD (NCT04127578), refractory epilepsy (NCT05613166) multiple system atrophy (MSA) [33], (NCT03589976), diabetic retinopathy (NCT00711490), diabetic macular edema (NCT00401115 and NCT00656643), Sturge-Weber syndrome (NCT03047980, NCT02080624), TSC (NCT05104983, NCT04595513, NCT02061397, NCT01929642, and NCT00490789), Lysosomal diseases (NCT03952637), Leigh syndrome (LS) (NCT03747328), Gaucher disease type 2 (NCT04411654), alcohol use disorder (NCT03732248), smoking cessation (NCT04161144), depression (NCT02487485), stroke prevention (NCT04948749), myalgic encephalomyelitis/chronic fatigue syndrome (ME/CFS) (NCT06257420), communicating hydrocephalus secondary to ICH (NCT06563817), post-traumatic stress disorder (PTSD) (NCT01449955), surgically refractory epilepsy (NCT03646240), extracranial vertebral artery stenosis (NCT05885932), ischemic stroke (IS) (NCT02578069), intracranial atherosclerotic stenosis (NCT05719883, NCT06614972, and NCT04949880), and mild cognitive impairment (NCT04200911).

Overall, in the context of clinical trials, four of them have successfully completed phase I (NCT00401115, NCT03732248, NCT03646240, and NCT04200911). Fourteen trials have advanced to complete phases I/II or II (NCT01445548, NCT02357342, NCT02732899, NCT00766649, NCT00304954, NCT02080624, NCT01929642, NCT00656643, NCT04161144, NCT02487485, NCT02061397, NCT00711490, NCT03359538, and NCT04595513). One trial has successfully concluded in phase II/III (NCT03047980), six trials have been withdrawn or terminated during phases I/II (NCT00095329, NCT01675947, NCT03589976, NCT00712491, NCT00766337, and NCT03747328), two trials are currently in phase II with an unknown status (NCT00490789, NCT05613166). As a TK inhibitor, sunitinib has been evaluated in two completed phase I/II trials targeting neovascular age-related macular degeneration (NCT03249740) and diabetic macular edema associated with retinal vein occlusion (NCT04085341). Temsirolimus, known for being a prodrug of sirolimus, inhibits the mTOR signaling pathway, has a completed phase II trial for the treatment of relapsing-remitting MS (NCT00228397). Tofacitinib, known for its role as a Janus kinase enzyme inhibitor, has been involved in multiple clinical trials. one of which is an ongoing phase I trial for MG patients (NCT04431895). An early phase I clinical trial (NCT06689982) explored the potential of tofacitinib in treating ALS and also a terminated phase I/II clinical trial of tofacitinib focused on treatment-resistant depression (NCT04141904). Another completed phase II trial focused on the application of tofacitinib in individuals with Down syndrome (DS) to address various skin conditions, including alopecia areata, atopic dermatitis/eczema, and psoriasis (NCT04246372). Trametinib, a MEK inhibitor, was used in a terminated phase I/II clinical trial for ALS (NCT04326283). Upadacitinib, a JAK1 selective inhibitor, is currently going through a phase III trial investigating its use in the treatment of giant cell arteritis (NCT03725202), comparing the efficacy of upadacitinib plus corticosteroids versus corticosteroids alone. Zanubrutinib, a BTK inhibitor, is being tested in an ongoing phase II trial (NCT05356858) for the treatment of NMOSD, a medical condition characterized by the immune system’s attack on the optic nerves and spinal cord. The clinical trial demonstrated that BTK activity is increased in both B cells and microglia, indicating that this medication plays a role in improving the pathology associated with NMOSD [34].

## Off-label use of FDA-approved kinase inhibitors for neurological treatment

Although the FDA has not approved any kinase inhibitors for the treatment of neurological disorders, several FDA-approved kinase inhibitors have been used off-label. For example, Everolimus has been documented for the treatment of patients with TSC-associated refractory seizures [35], epilepsy [36], hemimegalencephaly-related epilepsy [37], pediatric TSC-related epilepsy [38], and FCD [39]. Ibrutinib has been used for the treatment of patients with white matter injury from graft-versus-host disease (GVHD) [40], anti-myelin-associated glycoprotein neuropathy [41], and MS [42], while Imatinib has been used for the treatment of patients with BBB malfunction in DiGeorge syndrome [43], and in IS [44]. With mixed results, ruxolitinib and baricitinib were recently used in the treatment of AGS, an immune-mediated neurological disease refractory to conventional immunosuppression [45]. Tofacitinib has also been reported in the treatment of refractory autoimmune encephalitis, again with mixed results [46].

## Preclinical trials of FDA-approved kinase inhibitors for neurological treatment

A total of 20 FDA-approved kinase inhibitors have undergone evaluation in clinical trials focused on neurological disorders; however, there exists a significant number of preclinical investigations examining the impact of these FDA-approved kinase inhibitors on neurological conditions. Research has been conducted on abemaciclib in preclinical settings to explore its potential for treating motor neuron degeneration [47], PTSD [48], and AD [49, 50]. Abrocitinib, an inhibitor of JAK1, JAK2, JAK3, and Tyk2, has been investigated for its neuroinflammation and neuroprotective effects on TBI [51]. In preclinical studies, Afatinib has been assessed for its potential to treat neuroinflammation resulting from a lack of oxygen and glucose [52], MS [53], nicotine dependence [54], TSC [54], and cortical brain injuries [55]. The efficacy of Axitinib has been investigated as a possible treatment option for AD [56], IS [57, 58], and diabetes-induced neuropathy [59]. The potential of Alectinib as a therapeutic intervention for binge drinking has been explored [60, 61] and IS [62]. Preclinical studies have investigated the use of baricitinib as a potential therapeutic option for neurocognitive disorders resulting from HIV infection [63], encephalitis [64, 65], MS [65], hypersensitivity in DS [66], SCI [67], Hutchinson-Gilford progeria [68], AD [69–71], AGS [72–75], ALS [76], neuropathic pain [77], type 1 diabetes mellitus peripheral neuropathy and bone denervation [78] and chronic itching induced by frontal cortex neurons [79]. Research conducted in a preclinical setting suggests that Binimetinib could be a viable treatment for specific variants of AD [80]. The efficacy of Bosutinib has been investigated as a possible treatment option for ICH [81], cerebral ischemia [82], PD [83–86], AD [87], transactive response DNA binding protein 43 (TDP-43) pathology [88], stress-activated protein kinase interacting protein 1 (SIN1)-mediated neurotoxicity [89], botulinum neurotoxins [90], LBD [91, 92], and ALS [93–95]. Cabozantinib has undergone evaluation for its potential use in treating Rett syndrome (RTT) [96], and AD [97–99]. Crizotinib has undergone evaluation for its potential use in treating PD [100], AD [101], persistent pain [102], Toxoplasma gondii [103], and the condition known as craniosynostosis, which is associated with Saethre-Chotzen syndrome, involves the early closure of sutures in the skull, potentially resulting in abnormal head shapes and neurological complications [104].

Research has been conducted on Dabrafenib to assess its viability as a treatment of IS [105], SCI [106], PD [107, 108], ataxia caused by neurohistiocytosis of the cerebellum [109], and brain arteriovenous malformation (BAVM) [110]. Dasatinib has been tested for the potential treatment of lipopolysaccharide (LPS)-induced neuroinflammation [111], kainic acid-induced neuroinflammation [112], glaucoma [113], tau-associated pathology [114], MS [115], ALS [116–118], PD [117], cognitive dysfunction [119, 120], obesity-induced anxiety [121], chronic unpredictable stress that can induce cognitive deficits [122], fetal alcohol syndrome [123], botulinum neurotoxins [90], AD [124], endotoxemia [125], and IS [126]. In combination with quercetin, Dasatinib has been reported for treatment of tauopathy [127], seizure [128, 129], cognitive deficits [130, 131], TBI [132], AD [133–137], TBI [138], motor neuron disease [118], and brain aging models [139–143]. Deucravacitinib, a selective Tyk2 inhibitor, has been proven to inhibit monocyte activation [144], which may be useful in treating neuroinflammation. Research has been conducted on Duvelisib to assess its effectiveness in addressing peripheral neuropathy caused by Paclitaxel [145]. Erlotinib has undergone evaluation for its potential use in treating nerve fiber damage [146], intracranial aneurysm formation [147], ALS [148], diabetic peripheral neuropathy [149, 150], and memory loss [151]. The efficacy of Everolimus has been investigated as a possible treatment for encephalopathy of prematurity [152], atherosclerosis-associated brain hypoxia [153], IS [154–156], AD [157, 158], HD [159, 160], vascular dementia [161], LPS-induced neuroinflammation [162], insulin-dysfunction-related cognitive dysfunction [163], glutamate-induced neurotoxicity [164], Guillain-Barre’ syndrome [165], MS [166], TSC-associated autism-like social deficits [167, 168], Lafora disease [169], TSC-related epileptic encephalopathy [170–173], ICH [174], white matter injury [175], and hyperthermia-induced seizures [176] and neuroinflammation associated with seizures [177, 178].

Fedratinib has been evaluated for the potential treatment of IS [179], ICH [180], Wernicke’s encephalopathy [181, 182], and AD [183]. Fostamatinib was recently examined in experimental models for the treatment of schizophrenia [184] and ALS [185]. As a highly selective inhibitor, Fruquintinib specifically targets the TKs VEGFR 1, VEGFR 2, and VEGFR 3, has been carried out to assess the potential treatment options for cerebral amyloid angiopathy (CAA) [186]. The efficacy of gefitinib has been investigated as a possible treatment for SCIs [187], amyloid-β-induced memory loss [151], schizophrenia [188], *Streptococcus pneumoniae* meningitis [189], Toxoplasma gondii (that is capable of inducing symptoms that are characteristic of congenital neurological conditions and meningoencephalitis) [103, 190], IS [191], AD [192], and epilepsy [193].

The efficacy of Ibrutinib has been assessed in relation to the treatment of IS [194, 195], SCI [196, 197], age-related cognitive deterioration [198], AD [98, 199, 200], LPS-induced neuroinflammation [201], anxiogenic behavior [202], depression [203, 204]. Cocaine use disorder [205], SAH [206], and ALS [207]. Imatinib has been tested for the potential treatment of SAH [208–213], ICH [214–218], cerebral small vessel disease [219], TBI-induced seizure [220], seizures [220–222], TBI [223], IS [224–226], AD [227–244], PD [245–250], prion diseases [251–254], ALS [255], HD [256], cerebral malaria [257], Hypoxic ventilatory depression (that is characterized by a diminished ventilatory response triggered by hypoxemia, or low oxygen levels in the blood) [258], Niemann-Pick type C disease [259], Niemann-Pick type A disease [260], Gaucher disease [261], simian HIV encephalitis [262], morphine tolerance [263], BBB dysfunction in chronic cerebral hypoperfusion [264], and glutamate-induced oxidative injury [265]. Infigratinib has been evaluated as a prospective treatment option for diabetic retinopathy [266] and MS [267].

Lapatinib has been assessed for the potential treatment of epilepsy [268], organophosphate induced axonal damage in the spinal cord (it highlights the potential for neurotoxic effects that can compromise motor function and overall neural health) [269], AD [270, 271], PD [272], and BBB disruption in SARS-CoV-2 [273]. The efficacy of Lorlatinib has been investigated as a possible treatment option for persistent pain [102]. Midostaurin has been assessed for the potential treatment of traumatic SCI [274] and AD [275]. Neratinib has been tested for the potential treatment of AD [276, 277]. Netarsudil has been tested for the potential treatment of optic nerve degeneration [278]. Nilotinib has been tested for the potential treatment of Epileptic seizures [279], tauopathies [83, 84, 280], alpha-synucleinopathies [84, 281–283], TDP-43 pathology [85, 88], Beta-amyloid pathology [280], AD [69, 284–286], PD [287–289], chorea-acanthocytosis [290, 291], Niemann-Pick type A disease [260], LPS-induced cognitive impairment and neuroinflammation [292].

Pacritinib, an inhibitor of JAK1, JAK2, JAK3, and Tyk2, has been used to explore the potential interventions for RTT [293], HIV-associated neurological disorders, and also neurodegenerative diseases [294]. The efficacy of Palbociclib has been investigated as a possible therapeutic option for Spinal Muscular Atrophy (SMA) [295], amyloid beta-peptide pathology [296], and PD [297]. Research has been conducted on pazopanib to assess its efficacy in the treatment of tauopathy [298], neurodegeneration-induced memory and cognitive deficits [299], osteoarthritis pain [300], PD [301, 302], and AD [303]. Pexidartinib has been tested for the potential treatment of ICH [304–306], SAH [307], obesity-related cerebrovascular dysfunction [308], cognitive decline due to brain damage [309], tauopathy [310], AD [311, 312], HD [313], MS [314–316], spinocerebellar ataxia type 1 (SCA1) that is a genetic, neurodegenerative disorder that primarily affects the cerebellum [317], DS (also known as trisomy 21) [318], peripheral nerve injury induced mechanical hypersensitivity (also known as allodynia or hyperesthesia) [319], cocaine addiction [320], PD [321–323], psychiatric disorders like schizophrenia [324], cerebellar ataxias [325], LS [326, 327], opioid use disorder [328], psychiatric disorders such as depression [329], TBI [330], and neuropathic pain [331]. Ponatinib has been assessed for the potential treatment of IS [332], epilepsy [333], and cerebral cavernous malformation (abnormal blood vessel clusters within the brain may result in various neurological manifestations, including seizures, headaches, and the potential for stroke) [334]. Regorafenib has been assessed for the potential treatment of AD [335]. The efficacy of Ruxolitinib has been investigated as a possible treatment option for PD [336], MS [337–339], DS [340], dysfunction of the BBB induced by cytokines [341], HIV-related neurocognitive disorders [342], depressive-like behaviors and cognitive impairments [343], TBI [344, 345], IS [346], SCI [347, 348], AD with TDP-43 inclusions [70], and COVID-19 induced peripheral and CNS disease [349].

Selumetinib has been tested for the potential treatment of frontotemporal lobar degeneration [350], obsessive-compulsive disorder [351], acrolein-induced neurotoxicity [352], and ICH [353]. Sirolimus has been assessed for the potential treatment of IS [154, 354–388], TBI [389–396], SAH [397–403], SCI [404–415], germinal matrix hemorrhage (GMH) [416, 417], ICH [418–420], seizure-induced memory deficits [421–424], seizure in LS [425], spinal cord ischemia [426, 427], preganglionic cervical root transection [428], optic nerve crush [429], alveolar nerve transection [430], ischemic retinal disease [431], MS [432–442], PD [443–446], cerebral palsy (CP) [447], prion disease [448–450], AD [451–455], vascular dementia (that is a type of cognitive impairment that occurs due to reduced blood flow to the brain) [456], diabetes-related pathology that mimics AD [457], diabetes-induced neuropathology [458], HD [459–464], macular degeneration [465], optic nerve degeneration [466], neurodegeneration of retina [467], cadmium-induced neurodegeneration [468–471], spiral ganglion neurons degeneration [472], tauopathy [473, 474], synucleinopathy [475–477], MG [478, 479], iron-induced cognitive impairments [480], cognitive impairments resulting from intermittent hypoxia [481], cognitive impairments resulting from cannabinoid exposure [482], diabetic perioperative neurocognitive disorders [483], ethanol-induced neurodegeneration [484], neurodegeneration associated with aging [485], methylmercury-induced neurotoxicity [486], TDP-43 proteinopathy [487], ALS [488], autism spectrum disorder (ASD) [489–496], autism-associated behavioral disorders [497], Krabbe disease [498], trisomy 21 [499–502], intellectual disability [503], fetal alcohol spectrum disorders [504–506], autism linked to TS [507–510], TSC [511–514], TSC-associated neuropsychiatric disorders (TAND) [515], neurodevelopmental defects in TSC [516, 517], cognitive deficits in TSC [518, 519], Koolen-de Vries syndrome (that is a hereditary condition marked by delays in development, cognitive impairments, and unique facial characteristics) [520], FCD [521], Schaaf-Yang syndrome [522], cerebral malaria [523–525], neuropathic pain [526], seizure-induced anxiety [527], anxiety disorders [528–530], obesity-induced anxiety and depression [531], diabetes mellitus-related cognitive deficits [532–534], nicotine addiction [535], alcohol-related disorders [516, 536, 537]. Herpes simplex virus encephalitis [538], mania [539], epilepsy [172, 540–543], epilepsy-induced anxiety and depression [544], porcine hemagglutinating encephalomyelitis virus [545], photochemical damage in retinal photoreceptor cells [546], multisystem proteinopathy [547], N-methyl-D-aspartate (NMDA)-induced retinal damage [548–550], adverse optineurin phenotypes [551], hydrocephalus [552], sleep disorders [553], depression [554], drug-seeking behavior [555–557], Inflammation of the nervous system resulting from aging [558], mitochondrial encephalopathy [559], TANC2 mutation-induced neuropsychiatric disorders [560], impact of general anesthesia on neurodevelopmental disorders in individuals with fragile-X syndrome [561]. The behavioral manifestations of depression and anxiety induced by H. pylori infection [562], epileptic brain injury [563], age-related hearing loss [564], MSA [565], brain hypervascularization [566], sepsis-induced cognitive impairment [567], polyhydramnios, megalencephaly, and symptomatic epilepsy syndrome [568].

FCD type II induced epileptic seizures [569]. Sorafenib has been tested for the potential treatment of SAH [570], IS [571], SCI [572], AD [97, 573, 574], PD [575], MS [576, 577], rabies [578], Rift Valley fever (RVF) virus [579], alphaviruses [580], and picornavirus enterovirus 71 [581]. Sunitinib has been tested for the potential treatment of TBI [582], seizure [583], AD [584–587], RTT [96], cognitive impairment associated with HIV [588, 589], Dengue virus [590], rabies [591], and optic nerve injury [592].

Temsirolimus has been assessed for the potential treatment of SCI [410], PD [593, 594], tauopathy [595, 596], AD [597], SCA3 [598], nicotine withdrawal-associated cognitive deficits [599], and X-linked adrenoleukodystrophy (a genetic disorder primarily affecting the nervous system and adrenal glands) [600]. Tofacitinib has been evaluated for the potential treatment for IS [601], AD [602], MS [603, 604], PD [605], ALS [606], Venezuelan equine encephalitis virus (VEEV) [607], SCI [608], neuropsychiatric lupus (NPSLE) [609]. Trametinib has been tested for the potential treatment of TBI [610], aneurysmal SAH [611], BAVMs [612], ALS [613, 614], AD [615, 616], demyelinating disease [617]. Vandetanib has been tested for the potential treatment of GMH [618]. Vemurafenib has been tested for the treatment of laminin-α2-related congenital muscular dystrophy (LAMA2-CMD), which is a neuromuscular disease (LAMA2-CMD) [619].

## Structure modification for new chemical entities with improved BBB penetration

While repurposing FDA-approved kinase inhibitors for CNS disorders accelerates the clinical trials, early kinase inhibitors, which were often not developed to treat CNS disorders, exhibit limited selectivity and off-target side effects. Therefore, developing new BBB-penetrating chemical entities such as kinase inhibitors will provide a more targeted treatment of kinase-related CNS disorders. Because of the unique structure and function of the BBB, such as the tight junction and efflux pumps, small-molecule CNS drugs usually possess different structural features from non-CNS drugs from the medicinal chemistry perspective. For example, other than using Lipinski’s guidelines to design oral small-molecule drugs, Pfizer chemists have provided a more focused CNS multiparameter optimization (MPO) score to guide BBB-penetrating small-molecule discovery [620, 621]. The CNS MPO score evaluates a small molecule’s properties of cLogP (preferably < 3), cLogD (< 2), molecular weight (< 360), pKa (< 8), topological polar surface area (between 40 to 90), and the number of hydrogen-bond donors (< 2). Usually, a CNS MPO score of four or above indicates a higher probability of BBB penetration. It is a common strategy in drug discovery to improve the physiological properties of existing non-BBB-penetrating drugs by structural modifications so that the new chemical entities possess better BBB permeability. In this way, a therapeutic concentration can be reached in the CNS while the plasma concentration is maintained relatively low. In the next paragraph, we highlight some recent developments in CNS-penetrating kinase inhibitors that originate from FDA-approved drugs.

Erlotinib, a first-generation EGFR inhibitor with a low BBB penetration and an unbound brain-to-plasma partition coefficient (K_p,uu,brain_) of 0.05, was approved in the US to treat non-small cell lung cancer and pancreatic cancer. Structural optimization of erlotinib led to the discovery of JCN037 which has substantially improved BBB penetration with a K_p,uu,brain_ of 1.30 [622]. The structural modifications include rigidification of the molecular architecture by shortening the water-exposed ether chain of erlotinib as well as replacing the alkyne group with a bromine atom to improve the BBB-penetration. Ibrutinib, the first FDA-approved BTK inhibitor for the treatment of multiple cancers, such as leukemia and lymphoma, cannot accumulate in the CNS due to drug efflux by P-glycoprotein (P-gp) [623]. However, structural modifications have resulted in a new molecule, tolebrutinib [624], which can effectively penetrate the BBB. The major structural difference between Ibrutinib and tolebrutinib is the hinge-binding group: Ibrutinib utilizes a pyrimidine substructure, and tolebrutinib employs a more lipophilic pyridine substructure. Additionally, the FDA-approved BRAF^V600E^ inhibitor vemurafenib cannot cross the BBB, but after removing the flexible 4-chlorophenyl group and reducing the molecular weight, a new BRAF^V600E^ inhibitor, PF-07284890 (ARRY-461), was developed with improved BBB penetration [625]. Its phase Ia/b clinical trial (NCT04543188) was terminated recently due to an internal business decision, but not major safety concerns or requests from any regulatory authorities. Lastly, AZD0156, an ataxia-telangiectasia mutated (ATM) kinase inhibitor, shows an efflux ratio of 23.7 in MDCKII-MDR1-BCRP assays and is unlikely to penetrate the BBB. By increasing the lipophilicity of AZD0156, a new compound, AZD1390, exhibited substantially increased BBB penetration with an efflux ratio of 1.8 in MDCKII-MDR1-BCRP assays, unlikely to engage the efflux pump [626].

## Conclusions

By the end of October 2024, nearly all 87 FDA-approved kinase inhibitors have been tested in various animal models of non-cancerous neurological disorders, with twenty entered into clinical trials and six used for off-label treatments for non-cancerous neurological disorders in humans. More kinase inhibitors are expected to enter the pipeline of clinical trials for neurological indications. As compared to the new drugs that typically retain full IP rights and remain costly, repurposing kinase inhibitors, if proven successful, could offer effective and affordable treatments for non-cancerous neurological disorders.

## Supplementary Material

Other supplementary material for this article is available at: https://www.explorationpub.com/uploads/Article/file/1008116_sup_1.xlsx.

## Data Availability

Not applicable.

## Abbreviations

ACCD: Aberrant Cell Cycle Disease
AD: Alzheimer’s disease
AGS: Aicardi-Goutieres syndrome
ALS: amyotrophic lateral sclerosis
BAVM: brain arteriovenous malformation
BBB: blood-brain barrier
BTK: bruton tyrosine kinase
CNS: central nervous system
DS: Down syndrome
FCD: focal cortical dysplasia
GMH: germinal matrix hemorrhage
HD: Huntington’s disease
HIV: human immunodeficiency virus
ICH: intracerebral hemorrhage
IP: intellectual property
IS: ischemic stroke
K_p,uu,brain_: unbound brain-to-plasma partition coefficient
LAMA2-CMD: laminin-α2-related congenital muscular dystrophy
LBD: Lewy body dementia
LPS: lipopolysaccharide
LS: Leigh syndrome
MAPKK4/MEK4: mitogen-activated protein kinase kinase 4
MG: myasthenia gravis
MPO: multiparameter optimization
MS: multiple sclerosis
MSA: multiple system atrophy
NMOSD: neuromyelitis optica spectrum disorders
PD: Parkinson’s disease
PTSD: post-traumatic stress disorder
RTKs: receptor tyrosine kinases
RTT: Rett syndrome
SAH: subarachnoid hemorrhage
SCA1: spinocerebellar ataxia type 1
SCI: spinal cord injury
STKs: serine-threonine kinases
TBI: traumatic brain injury
TDP-43: transactive response DNA binding protein 43
TKs: tyrosine kinases
TSC: tuberous sclerosis complex
